# Identification of compound mutations of *SLC12A3* gene in a Chinese pedigree with Gitelman syndrome exhibiting Bartter syndrome-liked phenotypes

**DOI:** 10.1186/s12882-020-01996-2

**Published:** 2020-08-05

**Authors:** Bingzi Dong, Ying Chen, Xinying Liu, Yangang Wang, Fang Wang, Yuhang Zhao, Xiaofang Sun, Wenjuan Zhao

**Affiliations:** 1 Department of Endocrinology and Metabolism, the Affiliated Hospital of Qingdao University, 16 Jiangsu Road, Qingdao, 266003 China; 2 Department of Endocrinology, Pingdu People’s Hospital, 112 Yangzhou Road, Pingdu, 266700 China

**Keywords:** Hypokalemia, Gitelman syndrome, *SLC12A3*, Hypercalciuria

## Abstract

**Background:**

Gitelman syndrome is a rare salt-losing renal tubular disorder associated with mutation of *SLC12A3* gene, which encodes the Na-Cl co-transporter (NCCT). Gitelman syndrome is characterized by hypokalemia, metabolic alkalosis, hypomagnesemia, hypocalciuria, and renin-angiotensin-aldosterone system (RAAS) activation. Different *SLC12A3* variants may lead to phenotypic variability and severity.

**Methods:**

In this study, we reported the clinical features and genetic analysis of a Chinese pedigree diagnosed with Gitelman syndrome.

**Results:**

The proband exhibited hypokalaemia, hypomagnesemia, metabolic alkalosis, but hypercalciuria and kidney stone formation. The increased urinary calcium excretion made it confused to Bartter syndrome. The persistent renal potassium wasting resulted in renal tubular lesions, and might affect urinary calcium reabsorption and excretion. Genetic analysis revealed mutations of *SLC12A3* gene with c.433C > T (p.Arg145Cys), c.1077C > G (p.Asn359Lys), and c.1666C > T (p.Pro556Ser). Potential alterations of structure and function of NCCT protein due to those genetic variations of *SLC12A3* are predicted. Interestingly, one sibling of the proband carried the same mutant sites and exhibited similar clinical features with milder phenotypes of hypokalemia and hypomagnesemia, but hypocalciuria rather than hypercalciuria. Family members with at least one wild type copy of *SLC12A3* had normal biochemistry. With administration of spironolactone, potassium chloride and magnesium supplement, the serum potassium and magnesium were maintained within normal ranges.

**Conclusions:**

In this study, we identified compound mutations of *SLC12A3* associated with varieties of clinical features. Further efforts are needed to investigate the diversity in clinical manifestations of Gitelman syndrome and its correlation with specific *SLC12A3* mutations.

## Background

Gitelman syndrome (OMIM#263800) is an autosomal recessive inherited salt-losing renal tubular disorder. Gitelman syndrome is characterized with clinical features including hypokalemia, renal potassium wasting, metabolic alkalosis, hypomagnesemia, hypocalciuria, and RAAS activation with normal blood pressure [1]. Gitelman syndrome is associated with mutations of *SLC12A3* (solute carrier family 12 member 3) gene, which locates on chromosome 16q13 and encodes the thiazide-sensitive Na-Cl cotransporter (NCCT) of distal convoluted tubule (DCT) [2]. Till now, there are more than 400 varieties of *SLC12A3* related to Gitelman syndrome have been reported [2–5]. Among those mutations, most are missense, deletion, insertion, and splice-site mutations [6]. Most *SLC12A3* mutations in Gitelman syndrome are found as simple or complex heterozygous mutations, and few of them are homozygous [7].

The clinical symptoms of Gitelman syndrome are variable, including muscle weakness, paresthesia, numbness, polyuria, and growth retardation in children [2]. Some patients are asymptomatic or mildly symptomatic, or only exhibit non-specific fatigue, leading to the frequent misdiagnosis. Disordered renal reabsorption of sodium and chloride leads to a series of pathophysiological changes and clinical manifestations, including decreased blood volume, and activated renin-angiotensin aldosterone system (RAAS). Severe and persistent hypokalemia may lead to glucose intolerance, cardiac and renal dysfunction. Bartter syndrome is the most important genetic disorder to consider in the differential diagnosis of Gitelman syndrome, since both exhibit hypokalemia, metabolic alkalosis, and increased plasma renin activity and aldosterone levels with normal blood pressure. Such similar phenotypes as those diseases share can make differential diagnose challenging; however, urinary calcium excretion is often considered to be an important clue to distinguish these two disorders [8].

In this study, we reported a Chinese familial Gitelman syndrome, and identified compound mutations of *SLC12A3* with c.433C > T (p.Arg145Cys), c.1077C > G (p.Asn359Lys), and c.1666C > T (p.Pro556Ser). The proband presented hypercalciuria and renal calcification, exhibiting Bartter syndrome-like biochemical phenotypes. Further analysis of genotype-phenotype correlation analysis is needed to provide deeper insights into Gitelman syndrome.

## Methods

### Patient recruitment

Participants were recruited from a Chinese pedigree. The diagnosis of Gitelman syndrome was based on clinical symptoms, biochemical parameters and genetic analysis of *SLC12A3* mutations. All participants denied a history of laxatives, diuretics, or other agents including insulin, β-receptor activator or Chinese herbal medicine. This study was approved by the ethics committee of the Affiliated Hospital of Qingdao University.

### Biochemistry tests

The peripheral blood sample and urine sample were collected. The blood and urine electrolytes were measured with an automatic biochemical analyzer. Plasma renin activity, plasma angiotensin, and plasma aldosterone were measured using a radioimmunoassay.

### Genetic analysis

Genomic DNA was extracted from peripheral blood samples collected from participants using the QIAamp Blood DNA Mini Kit (QIAGEN, USA) according to the manufacturer’s protocol. After amplification using 2X polymerase chain reaction (PCR) MasterMix polymerase (Tiangen, China) by ABI9700 PCR (Life technology, USA), the products were captured and purified with Panel probe (Illumine Inc., USA), then directly sequenced on the ABI 3500 automated DNA sequencer (Life technology, USA). Mutations were detected using next generation sequencing (NGS) and subsequently confirmed using Sanger sequencing. We investigated 40 genes reported to be associated with Gitelman syndrome or Bartter syndrome, including *CLCNKA, CLCNKB, BSND, LAMC, LAMB, ALMS, EDAR, CASR, DSP, BFSP2, TMEM67, PLEC, KIF7, SLC12A3, KRT9, KRT10, KRT14, PNPLA6* and so on. To assess the pathogenicity of the variants, those variants were analyzed with PolyPhen-2 (http://genetics.bwh.harvard.edu/pph2/), Mutation Taster (http://mutationtaster.org), PROVEAN and SIFT (http://provean.jcvi.org/) as alignment reference databases. Gene mutation databases such as 1000 Genomes, dbSNP, and Uniprot were used for reference genome alignment.

## Results

### Clinical manifestations of the proband

**Fig. 1:**
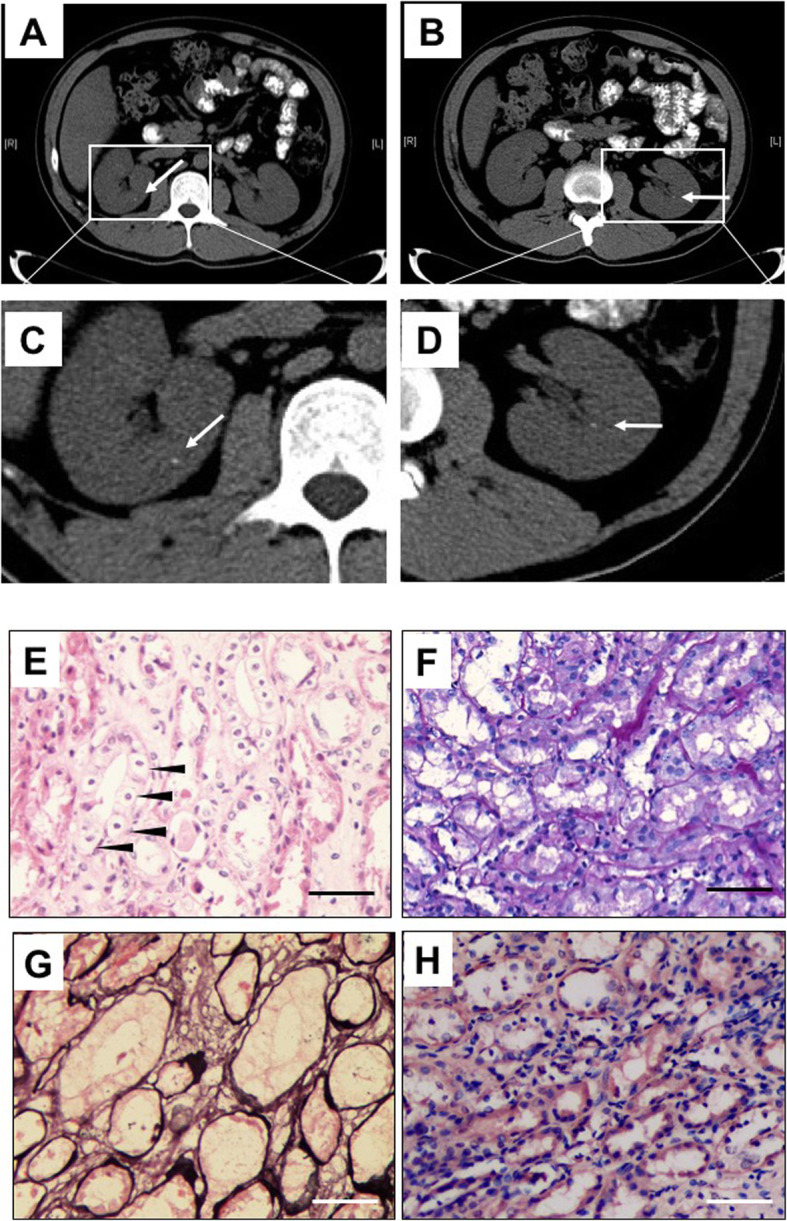
Imaging manifestations and renal biopsy to show renal lesions. **A-D** Computed tomography (CT) scan of the proband. Arrows to show renal calcification. C and D are the magnification of A and B, respectively. **E-H** Renal pathomorphism of the patient to show renal tubular lesions. E. Hematoxylin-eosin (HE) staining. F. Periodic acid Schiff (PAS) staining. G. Sliver methenamine (SM) staining. H. Congo red staining. Those show renal tubular atrophy, epithelial cell edema, and the thickening of basal membrane. The vacuolar degeneration of tubular epithelial cells and loss of brush border were observed. SM and Congo red staining were negative. (× 200) Arrowheads in panel E indicate degenerated tubular epithelial cells. The scale bar in panel E-H stands for 100 μm

A 42-year-old male was presented to hospital with chief complaint of fatigue, repeated muscle weakness and paralysis over ten-year period. Laboratory investigation exhibited hypokalemia, hypomagnesemia, increased urinary potassium excretion, activated RAAS, hypercalcemia and hypercalciuria (Table 1). The fractional excretion rate of potassium (FE_K_%) was significantly increased to 30.5–49.2% (normal range 8–12%), suggesting that hypokalemia is resulted from renal potassium loss. Thyroid function, cortical and adreno-corticotropic hormone (ACTH) were normal. Other possible causes of hypokalemia such as thyrotoxic periodic paralysis, renal tubular acidosis and hypercortisolism were excluded. Serum calcium was slightly increased, with elevated urinary calcium excretion (FE_Ca_ 2.66%, urinary calcium to creatine ratio 0.70) (Table 1). The glucose stimulated insulin secretion (GSIS) test showed the delayed insulin release and insulin resistance. Renal calcification was detected by computed tomography (CT) (Fig. 1A-D). Kidney biopsy revealed the large vacuolar degeneration or atrophy in renal tubular cells (Fig. 1E-H), suggesting that renal tubular lesions might be associated with persistent renal potassium wasting.

**Table 1:** Biochemical profiles and genetic variants of the Gitelman syndrome pedigree

		I-1	I-2	II-1	II-2	II-4	II-5	II-6	III-2	Normal
**Age/sex**		79 M	79F	55F	53 M	48F	47F	42 M	20 M	
**Biochemistry profile**										
Serum K	(mmol/L)	3.94	4.19	4.35	4.01	3.08↓	4.13	1.9–2.8↓↓	4.02	3.5–5.5
Serum Mg	(mmol/L)	0.99	0.85	0.99	0.95	0.68↓	0.97	0.71↓	0.98	0.8–1.02
Serum Ca	(mmol/L)	2.36	2.31	2.47	2.37	2.5	2.32	2.48–2.6	2.42	2.1–2.52
FE_K_	(%)	6.56	6.33	5.56	8.12	12.66↑	6.38	30.5–49.2↑↑	5.08	8–12%
uCa/Cr		0.29	0.30	0.34	0.19	0.03–0.07↓	0.28	0.7↑	0.33	
FE_Ca_	(%)	0.46	0.73	0.61	0.42	0.05–0.14↓	0.51	2.66↑	0.54	
RAAS (lying condition)										
Renin (ng/mL/hr)		–	–	–	–	2.47	–	3.58	–	0.15–2.3
Aldosterone (pg/mL)		–	–	–	–	79.53	–	123.72	–	30–160
RAAS (standing condition)										
Renin (ng/mL/hr)		–	–	–	–	9.36	–	> 13.56	–	0.1–6.5
Aldosterone (pg/mL)		–	–	–	–	143.5	–	279.65	–	70–300
**Genetic variants**		c.1077C > G	c.433C > T c.1666C > T	c.433C > T c.1666C > T	c.433C > T c.1666C > T	c.433C > T c.1077C > G c.1666C > T	c.433C > T c.1666C > T	c.433C > T c.1077C > G c.1666C > T	c.1077C > G	
		p.Asn359Lys	p.Arg145Cys p.Pro556Ser	p.Arg145Cys p.Pro556Ser	p.Arg145Cys p.Pro556Ser	p.Arg145Cys p.Asn359Lys p.Pro556Ser	p.Arg145Cys p.Pro556Ser	p.Arg145Cys p.Asn359Lys p.Pro556Ser	p.Asn359Lys

The patient was supplied with potassium chloride sustained release tables, spironolactone and magnesium. During the fellow-up, serum potassium and magnesium levels were maintained within normal range.

### Biochemistry profiles of the other pedigree relatives

**Fig. 2:**
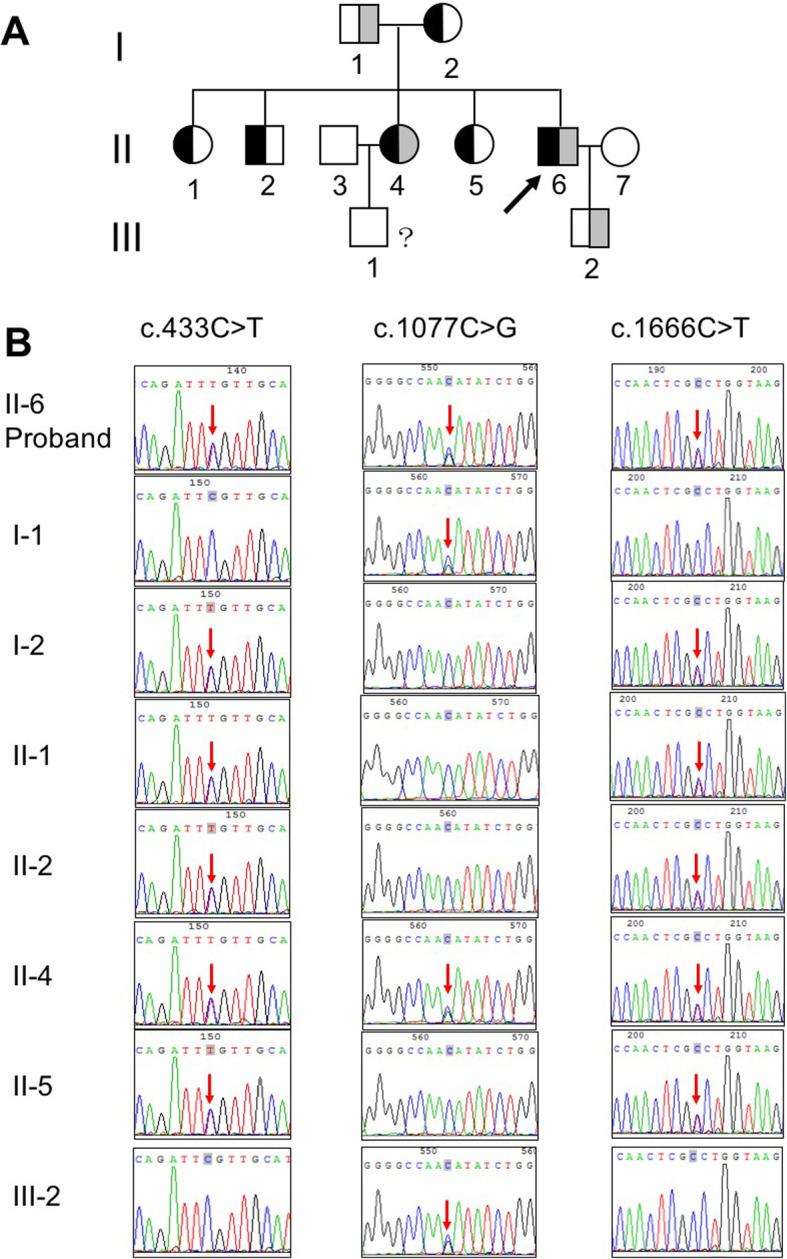
Genetic analysis of *SLC12A3* mutations in the pedigree of Gitelman syndrome. **A** Pedigree of the family structure. Marked symbols to show patients carried compound heterozygous mutations of *SLC12A3*. Mutations of c.433 C > T and c.1666 C > T was presented as black, and c.1077 C > G was showed as grey. Circles present females, and squares present males. Arrow shows proband. The III-1 and III-2 show normal phenotypes, without features of Gitelman syndrome. **B** Sequencing results of variants of *SLC12A3*. The patient (II-6, proband) and his mother (I-2), brother (II-2) and sisters (II-1; II-4; II-5) carried heterozygous mutation of C433T (Arg145Cys) and C1666T (Pro556Ser) in Exon 3 and 13 of *SLC12A3*, respectively. Heterozygous mutation of C1077G (Asn359Lys) in Exon 8 was detected in the patient (II-6, proband), his father (I-1), his son (III-2), and the sister with Gitelman syndrome (II-4). Arrows indicate heterozygous nucleotide substitutions

Plasma biochemical results showed that one of proband’s sister (II-4, Fig. 2A) showed Gitelman syndrome-phenotypes with hypokalemia, hypomagnesaemia, elevated renin-aldosterone level, and normal blood pressure, but with normocalcemia and hypocalciuria (Table 1). The sister was therefore administrated with potassium chloride, and the serum potassium level was corrected into normal range, without symptoms of fatigue, muscle weakness, tetany, or paresthesia during the treatment. The levels of serum potassium, sodium, calcium, magnesium, urinary potassium and calcium were unremarkable in the other relatives of the pedigree (Table 1).

### Genetic analysis

Sequencing of *SLC12A3* gene was performed on the familial relatives of the proband. Genetic analysis revealed that the proband and sibling II-4 carried same compound heterozygous mutations c.433C > T (p.Arg145Cys), c.1077C > G (p.Asn359Lys), and c.1666C > T (p.Pro556Ser). His father (I-1) and son (III-2) carried c.1077 C > G (p.Asn359Lys). His mother (I-2) and the other siblings (II-1, II-2, II-5) carried c.433 C > T (p.Arg145Cys) and c.1666 C > T (p.Pro556Ser), and showed phenotypically normal biochemistry (Fig. 2, Supplement figure, Tables 1 and 2). These results suggested that c.433C > T and c.1666C > T located on the same chromosome (in cis), while c.1077C > G representing the trans variant led to the compound heterozygosity in the proband and sibling (II-4). The frequency of these variants was examined in the reference database. The variant c.433C > T was not identified in 1000 Genomes, but was examined in gnomAD (0.000012) and Esp6500 (0.000077) at very low frequency. The variant c.1077C > G was identified in 1000 Genomes (0.0001997). The variant c.1666C > T was not identified in 1000 Genomes, but was examined in gnomAD at low frequency (0.00002). The potential pathogenicity of those three variants were studied using prediction bioinformatics (Table 2). In addition, no mutations were detected in other 40 genes reported to be associated with Gitelman and Bartter syndrome, including *CLCNKA/CLCNKB* (encodes the chloride channel ClC-Kb) and *BSND* (encodes chloride channel accessory subunit), *KCNJ1* (encodes the thick ascending limb potassium channel), and *CASR* (encodes Calcium-sensing receptor) were not detected.

**Table 2:** Summary of the variants of *SLC12A3* in the pedigree of Gitelman syndrome

Exon	Nucleotide mutations	Amino acid variants	Variant type	AF in 1000G	gnomAD_exome	Esp6500	PolyPhen-2	PROVEAN score	Mutation Taster	SIFT
3	c.433 C > T	p.Arg145Cys	missense	ND	0.000012	0.000077	0.999	−7.139 (Deleterious)	0.9999 (Disease)	0
8	c.1077 C > G	p.Asn359Lys	missense	0.00019	ND	ND	1.000	−5.706 (Deleterious)	0.9999 (Disease)	0
13	c.1666 C > T	p.Pro556Ser	missense	ND	0.00002	ND	0.331	−7.03 (Deleterious)	0.9999 (Disease)	0.002

*ND* not identified

### Three-dimensional structure prediction of NCCT and the potential dysfunction

**Fig. 3:**
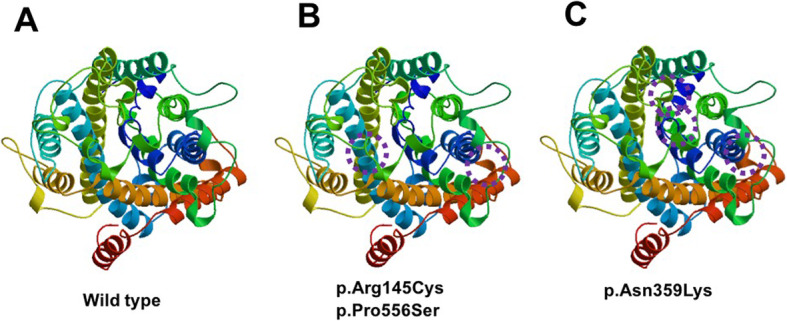
The model structure of Na-Cl cotransporter (NCCT) protein with variants induced by novel mutations of *SLC12A3* to show potential influence. The differences of modeled structure compared to wild type (**A**) were indicated in circles. The visible differences of protein structure was induced by (**B**) co-existence of c.433 C > T (p.Arg145Cys) and c.1666 C > T (p.Pro556Ser), or (**C**) c.1077 C > G (p.Asn359Lys) lead to differences from wild type protein structure. It may induce the alteration of the function of NCCT

The *SLC12A3*-encoded NCCT protein contains 12 transmembrane segments as well as N- and C-terminal domains. We identified the alteration of NCCT structure induced by the compound mutations of *SLC12A3* (C433T, Arg145Cys; C1077G, Asn359Lys; and C1666T, Pro556Ser) (Fig. 3), using the SWISS-MODEL workspace (http://swiss-model.expasy.org). Results indicate that amino acid change in NCCT protein due to the missense mutations of *SLC12A3* might lead to alteration of NCCT protein structures that can affect function, resulting in the electrolyte disturbance.

## Discussion

In this study, we reported a Chinese pedigree of Gitelman syndrome with heterozygous compound mutations of *SLC12A3*, exhibiting hypokalemia and hypomagnesemia. However, the proband exhibited hypercalciuria and renal calcification, which made it difficult to differentiate from Bartter syndrome (especially type III). We identified variants of *SLC12A3,* c.433C > T (p.Arg145Cys), c.1077C > G (p.Asn359Lys), and c.1666C > T (p.Pro556Ser), but no mutations found in *CLCNKA/CLCNKB*, *BSND, KCNJ1*, *CASR* that are known causing Batter syndrome. Therefore, clinical diagnosis of Gitelman syndrome was made, and the identified *SLC12A3* variations were predicted to contribute clinical features of Gitelman syndrome. With administration of aldosterone antagonist spironolactone, potassium and magnesium supplement, the serum potassium and magnesium was maintained in nearly normal range during fellow-up. The phenotype variability may be associated with the pathogenic variabilities of *SLC12A3* mutations. Genetic analysis is a useful tool for the diagnosis and differential diagnosis of such similarly-presenting diseases as Gitelman syndrome and Bartter syndrome. Further investigation is needed to provide better understanding of genotype-phenotype association of NCCT dysfunction in Gitelman syndrome.

Gitelman syndrome is a salt-losing tubulopathy with the clinical features of hypokalemic alkalosis, hypomagnesemia and hypocalciuria. Chronic hypokalemia leads to symptoms of weakness, fatigue, thirst, and paralysis. Severe cases can cause rhabdomyolysis, ventricular arrhythmias, or even sudden cardiac arrest [9]. Gitelman syndrome is associated with dysfunction of NCCT protein encoded by *SLC12A3* gene in the renal DCT. The decreased reabsorption of Na^+^ and Cl^−^ leads to compensatory excessive exchange through Na^+^/K^+^ and Na^+^/H^+^ pumps, resulting in excessive K^+^ and H^+^ excretion and hypokalemic alkalosis. In a small minority of Gitelman syndrome patients, mutations in the *CLCNKB* gene encoding the chloride channel ClC-Kb have been identified [10].

We identified compound mutations of *SLC12A3,* c.433C > T (p.Arg145Cys), c.1077C > G (p.Asn359Lys), and c.1666C > T (p.Pro556Ser). The proband and his affected sister carried three compound heterozygous mutations, suggesting that c.433 C > T and c.1666 C > T probability occurred *in cis* on one allele, and c.1077C > G occurred *in trans*. Interestingly, the other relatives with only a single affected chromosome show normal biochemistry. The phenotypes are more severe in patients with more than one mutated allele, with lower serum potassium level, which were more difficult to be corrected with potassium supplements [6, 11].

Bartter syndrome (especially type III) is the most important renal salt-wasting disease which should be considered as the differential diagnosis of Gitelman syndrome. Bartter syndrome is also characterized by hypokalemia, metabolic alkalosis, polyuria, increased renin activity and aldosterone levels, but without hypertension or edema. It exhibits the increased urinary calcium excretion, but rarely leads to nephrocalcinosis. Bartter syndrome could be caused by mutations of *NKCC2* (Na^+^-K^+^-2Cl^−^ cotransporter) expressed in the thick ascending limb (TAL) of Henle loop (Type 1 Bartter Syndrome) [12], *ROMK* (outwardly rectifying potassium channel) (Type 2 Bartter Syndrome), or *CLCNKB* (chloride channel) (Type 3 Bartter Syndrome) which is a regulator of NKCC2. Type 4 Bartter Syndrome is induced by mutations of both the kidney-specific chloride channel ClC-Ka and ClC-Kb, leading to dysfunction of Cl^−^ reabsorption. Activating mutations of calcium-sensing receptor (CaSR) suppresses the NKCC2 and ROMK expression to induce type 5 Bartter syndrome [13]. The site of defect in Bartter syndrome is at the TAL of the Henle loop, whereas in Gitelman syndrome is at the renal DCT [14]. Gitelman syndrome used to be thought as a mild type of Bartter syndrome. However, the pathogenesis and clinical characteristics are different. Bartter syndrome typically presents in infancy or early childhood, with more severe clinical manifestations and complications, such as severe electrolyte derangements, short stature, polyuria, and hypercalciuria induced nephrocalcinosis [15]. Gitelman syndrome usually shows hypomagnesaemia with increased urinary magnesium excretion (FE_Mg_ > 4%), but lower urinary calcium excretion (uCa/uCr < 0.2) [8]. A diuretic loading test using furosemide and hydrochlorothiazide can be helpful in differentiating Gitelman syndrome from Bartter syndrome [16].

Hypocalciuria in Gitelman syndrome is generally a result of the increased calcium reabsorption in the proximal tubule and distal renal unit, which is caused by NCCT dysfunction [17]. In this report, the proband exhibited hypokalemia, hypomagnesaemia, metabolic alkalosis, but with hypercalciuria, similar to the features of Bartter syndrome, which makes it confused for differential diagnosis. It is contradicted with the features of hypocalciuria in classic Gitelman syndrome. Chronic renal potassium loss can cause renal tubular epithelial cell injury or vacuolar deformation to reduce the reabsorption of calcium [18]. The persistent renal potassium wasting might result in renal tubular lesions, and affect urinary calcium reabsorption and excretion, leading to persistent hypercalcuria. Additionally, loss-of-function of NCCT up-regulates the expression of intestinal calcium transporter, and increases calcium uptake in digestive tract [19]. Hypercalcemia inhibits PTH release via negative feedback; conversely, the suppressed PTH level reduces the calcium reabsorption by the renal tubule, and increases urinary calcium excretion. The patient described here also has diabetes mellitus. Hyperglycemia causes osmotic diuresis leading to urinary calcium excretion. Finally, increased urinary calcium excretion and chronic hypomagnesaemia are the causes of renal calcification. The relationship between mutated gene sites and urinary calcium levels has not been reported. It is unclear whether hypercalcuria is associated with three variants of *SLC12A3*.

Patients with Gitelman syndrome have a tendency of glucose intolerance and impaired insulin secretion [20]. Potassium plays an important role in the regulation of insulin release. Reduced extracellular potassium ion concentration could suppress the insulin secretion and release via ATP-sensitive potassium channel on beta-cells. Long-term low potassium and magnesium level is one of the factors for diabetes development. In addition, hyperaldosteronism was also reported to promote insulin resistance [21]. Studies have indicated that Gitelman syndrome can be combined with autoimmune diseases such as Graves’ disease, Hashimoto’s thyroiditis, IgA nephropathy, Sjogren’s syndrome, or latent autoimmune diabetes in adults (LADA) [22, 23].

The therapeutic strategy for Gitelman syndrome focuses on the correction of electrolyte disturbance, especially potassium and magnesium replacement. The level of serum magnesium may affect the severity and effect of potassium supplement [6, 24]. Other therapeutic options include the inhibitors of the secondary elevated RAAS, such as using non-selective or selective aldosterone antagonist spironolactone or eplerenone, or NaCl transporter blockers such as potassium-sparing diuretic aminophenidine. The loss-of-function mutations in the SLC12A3 gene result in Gitelman syndrome. Thus, the thiazide diuretics should be avoided for Gitelman syndrome patients as long-term treatment option [25]. Non-steroidal anti-inflammatory drugs (NASIDs) such as indomethacin can suppress renin secretion by inhibiting renal prostaglandin E2 (PGE_2_) synthesis, and ameliorate the up-regulation of aldosterone level induced by potassium supplement. It also could increase potassium level without worsening sodium and volume depletion in Gitelman syndrome patients [26]. However, the gastrointestinal side effect and interstitial renal damage make the application to be limited.

## Conclusions

In this study, we reported a pedigree of Gitelman syndrome and identified compound mutations of *SLC12A3*. Combined with clinical features, biochemistry profiles and genetic analysis, the diagnose could be made. The proband exhibited persistent hypercalcuria, which is contrary to the typical biochemical alteration in Gitelman syndrome. Diagonotic procedures combined clinical features and biochemistry profiles with genetic counselling are necessary for correctly differentiate Gitelman syndrome from Bartter syndrome. Further investigation will explore the correlation between genotype and phenotype are needed for provide better understanding of Gitelman syndrome.

## Supplementary information

**Additional file 1. **The variants of *SLC12A3* identified in this pedigree of Gitelman syndrome. A. The mutant sequence of *SLC12A3* mRNA and amino acid. Red characters to show the mutant nucleotide or amino acid. B. The model of Na-Cl cotransporter (NCCT) and affected amino acid site. NCCT is a 12 times transmembrane structure with 1030 amino acids. Star and red to show the mutant amino acids and sites, Arg145Cys, Asn359Lys, and Pro556Ser located in the respective spots.

## Data Availability

The data generated and analyzed in this study are not publicly available due to protection of privacy, but are available from the corresponding author on reasonable request. The genetic sequencing data were deposited at NCBI under bioproject SRA accession number PRJNA647666 (http://www.ncbi.nlm.nih.gov/bioproject/PRJNA647666).

## Abbreviations

GS: Gitelman syndrome
NCCT: The Na-Cl co-transporter
RAAS: Renin-angiotensin-aldosterone system
BS: Bartter syndrome
PCR: Polymerase chain reactions
NGS: Next generation sequencing
FE_K_: Fraction excretion rate of potassium
ACTH: Adreno-corticotropic hormone
GSIS: Glucose stimulated insulin secretion
CT: Computed tomography
DCT: Distal convoluted tubule
NKCC2: Na^+^-K^+^-2Cl^−^ cotransporter
TAL: Thick ascending limb
CaSR: Calcium-sensing receptor
LADA: Latent autoimmune diabetes in adults
NASID: Non-steroidal anti-inflammatory drugs
PGE_2_: Prostaglandin E_2_

## References

[1] Knoers NV, Levtchenko EN (2008). Gitelman syndrome. Orphanet J Rare Dis.

[2] Riveira-Munoz E, Chang Q, Bindels RJ, Devuyst O (2007). Gitelman's syndrome: towards genotype-phenotype correlations?. Pediatr Nephrol.

[3] Riveira-Munoz E, Chang Q, Godefroid N (2007). Transcriptional and functional analyses of SLC12A3 mutations: new clues for the pathogenesis of Gitelman syndrome. J Am Soc Nephrol.

[4] Qin L, Shao L, Ren H (2009). Identification of five novel variants in the thiazide-sensitive NaCl co-transporter gene in Chinese patients with Gitelman syndrome. Nephrology..

[5] Luo JW, Meng XR, Yang X (2017). Analysis of mutations of two Gitelman syndrome family SLC12A3 genes and proposed treatments using Chinese medicine. Chin J Integr Med.

[6] Li C, Zhou X, Han W (2015). Identification of two novel mutations in SLC12A3 gene in two Chinese pedigrees with Gitelman syndrome and review of literature. Clin Endocrinol.

[7] Nakhoul F, Nakhoul N, Dorman E, Berger L, Skorecki K, Magen D (2012). Gitelman's syndrome: a pathophysiological and clinical update. Endocrine..

[8] Bettinelli A, Bianchetti MG, Girardin E (1992). Use of calcium excretion values to distinguish two forms of primary renal tubular hypokalemic alkalosis: Bartter and Gitelman syndromes. J Pediatr.

[9] Cruz DN, Shaer AJ, Bia MJ, Lifton RP (2001). Simon DB; Yale Gitelman’s and Bartter’s syndrome collaborative study group. Gitelman’s syndrome revisited: an evaluation of symptoms and health-related quality of life. Kidney Int.

[10] Cho HW, Lee ST, Cho H, Cheong HI (2016). A novel mutation of CLCNKB in a Korean patient of mixed phenotype of Bartter-Gitelman syndrome. Korean J Pediatr.

[11] Balavoine AS, Bataille P, Vanhille P (2011). Phenotype-genotype correlation and follow-up in adult patients with hypokalaemia of renal origin suggesting Gitelman syndrome. Eur J Endocrinol.

[12] Simon DB, Karet FE, Hamdan JM, DiPietro A, Sanjad SA, Lifton RP (1996). Bartter’s syndrome, hypokalaemic alkalosis with hypercalciuria, is caused by mutations in the Na-K-2Cl cotransporter NKCC2. Nat Genet.

[13] Shaer AJ (2001). Inherited primary renal tubular hypokalemic alkalosis: a review of Gitelman and Bartter syndromes. Am J Med Sci.

[14] Fremont OT, Chan JC (2012). Understanding Bartter syndrome and Gitelman syndrome. 2012. World J Pediatr.

[15] Matsunoshita N, Nozu K, Shono A (2016). Differential diagnosis of Bartter syndrome, Gitelman syndrome, and pseudo-Bartter/Gitelman syndrome based on clinical characteristics. Genet Med.

[16] Goswami RP, Mandal S, Karmakar PS, Ghosh A (2011). Diuretic loading test and use of Bartter's Normogram in diagnosing a case of Gitelman's syndrome: relook into pathophysiology. Indian J Nephrol.

[17] Nijenhuis T, Vallon V, van der Kemp AW, Loffing J, Hoenderop JG, Bindels RJ (2005). Enhanced passive Ca^2+^ reabsorption and reduced Mg2+ channel abundance explains thiazide-induced hypocalciuria and hypomagnesemia. J Clin Invest.

[18] Tseng MH, Yang SS, Hsu YJ (2012). Genotype, phenotype, and follow-up in Taiwanese patients with salt-losing tubulopathy associated with SLC12A3 mutation. J Clin Endocrinol Metab.

[19] Hsu YJ, Yang SS, Cheng CJ (2015). Thiazide-sensitive Na+−cl− Cotransporter (NCC) gene inactivation results in increased duodenal Ca2+ absorption, enhanced osteoblast differentiation and elevated bone mineral density. J Bone Miner Res.

[20] Ren H, Qin L, Wang W (2013). Abnormal glucose metabolism and insulin sensitivity in Chinese patients with Gitelman syndrome. Am J Nephrol.

[21] Zennaro MC, Caprio M, Fève B (2009). Mineralocorticoid receptors in the metabolic syndrome. Trends Endocrinol Metab.

[22] Mizokami T, Hishinuma A, Kogai T (2016). Graves' disease and Gitelman syndrome. Clin Endocrinol.

[23] Kim YK, Song HC, Kim WY (2008). Acquired Gitelman syndrome in a patient with primary Sjögren syndrome. Am J Kidney Dis.

[24] Jiang L, Chen C, Yuan T (2014). Clinical severity of Gitelman syndrome determined by serum magnesium. Am J Nephrol.

[25] Seyberth HW, Schlingmann KP (2011). Bartter- and Gitelman-like syndromes: salt-losing tubulopathies with loop or DCT defects. Pediatr Nephrol.

[26] Blanchard A, Vargas-Poussou R, Vallet M (2015). Indomethacin, Amiloride, or Eplerenone for treating hypokalemia in Gitelman syndrome. J Am Soc Nephrol.

