# Supplementary figures and images for: Identification of compound mutations of SLC12A3 gene in a Chinese pedigree with Gitelman syndrome exhibiting Bartter syndrome-liked phenotypes
Source: BMC Nephrol. 2020 Aug 5;21:328. doi: 10.1186/s12882-020-01996-2 (PMC7409507; doi:10.1186/s12882-020-01996-2)

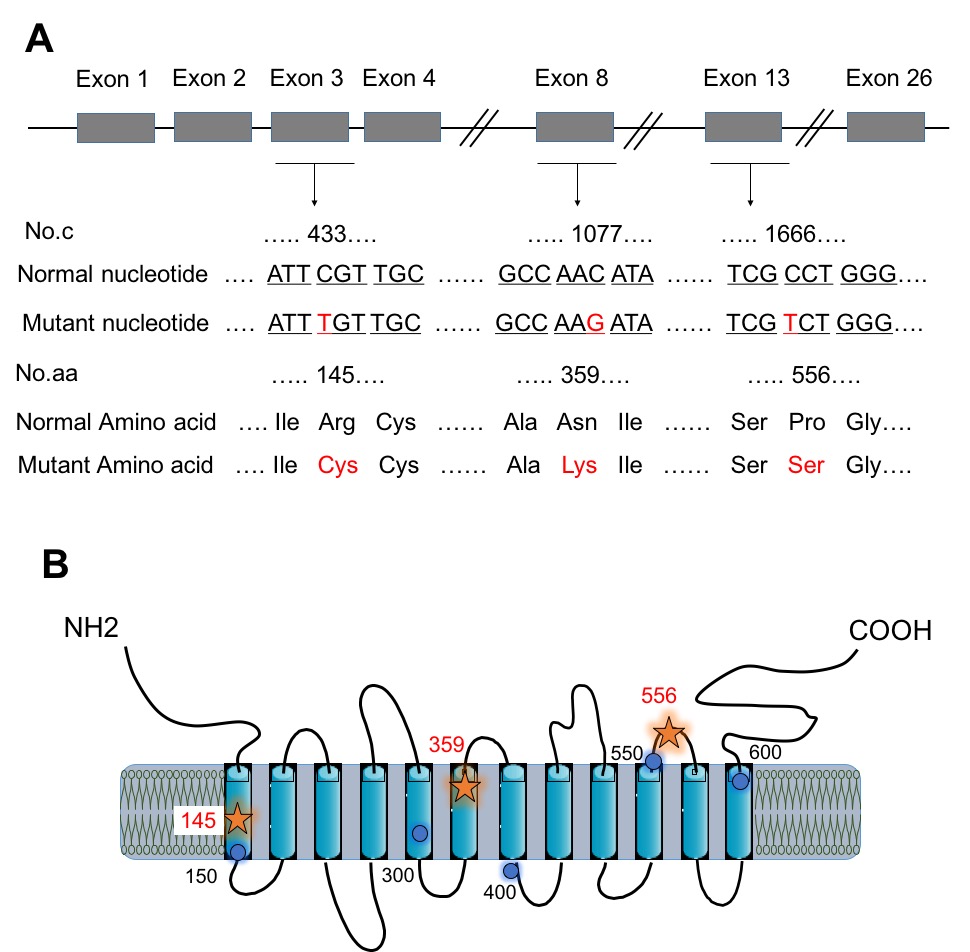

Supplement: Supplementary file 1 — Additional file 1. The variants of SLC12A3 identified in this pedigree of Gitelman syndrome. A. The mutant sequence of SLC12A3 mRNA and amino acid. Red characters to show the mutant nucleotide or amino acid. B. The model of Na-Cl cotransporter (NCCT) and affected amino acid site. NCCT is a 12 times transmembrane structure with 1030 amino acids. Star and red to show the mutant amino acids and sites, Arg145Cys, Asn359Lys, and Pro556Ser located in the respective spots. [file 12882_2020_1996_MOESM1_ESM.jpg]
